# Supplementary material for: Modifier Genes in Microcephaly: A Report on WDR62, CEP63, RAD50 and PCNT Variants Exacerbating Disease Caused by Biallelic Mutations of ASPM and CENPJ
Source: Genes (Basel). 2021 May 13;12(5):731. doi: 10.3390/genes12050731 (PMC8153008; doi:10.3390/genes12050731)

Supplementary Materials: Supporting data consists of supplementary method, three figures and one table.

Supplementary methods

Linkage analysis

Genome-wide linkage analysis was performed using the graphical user interface ALOHOMORA[1]. Relationship errors were identified by the program Graphical Relationship Representation [2]. The program PedCheck was applied to find Mendelian errors [3], and data for SNPs with such errors were removed from the data set. Non-Mendelian errors were identified by using the program MERLIN [4], and unlikely genotypes for related samples were deleted. Linkage analysis was performed assuming autosomal recessive inheritance, full penetrance, consanguinity and a disease allele frequency of 0.0001. Multipoint LOD scores were calculated using ALLEGRO [5]. Haplotypes were reconstructed with ALLEGRO and presented graphically with HaploPainter [6].

Supplementary References

1. Ruschendorf, F.; Nurnberg, P. ALOHOMORA: a tool for linkage analysis using 10K SNP array data. *Bioinformatics* **2005**, *21*, 2123-2125, doi:10.1093/bioinformatics/bti264.
2. Abecasis, G.R.; Cherny, S.S.; Cookson, W.O.C.; Cardon, L.R. GRR: graphical representation of relationship errors. *Bioinformatics* **2001**, *17*, 742-743.
3. O'Connell, J.R.; Weeks, D.E. PedCheck: A program for identification of genotype incompatibilities in linkage analysis. *Am. J. Hum. Genet.* **1998**, *63*, 259-266.
4. Abecasis, G.R.; Cherny, S.S.; Cookson, W.O.; Cardon, L.R. Merlin--rapid analysis of dense genetic maps using sparse gene flow trees. *Nat. Genet.* **2002**, *30*, 97-101, doi:10.1038/ng786.
5. Gudbjartsson, D.F.; Jonasson, K.; Frigge, M.L.; Kong, A. Allegro, a new computer program for multipoint linkage analysis. *Nat. Genet.* **2000**, *25*, 12-13.
6. Thiele, H.; Nurnberg, P. HaploPainter: a tool for drawing pedigrees with complex haplotypes. *Bioinformatics* **2005**, *21*, 1730-1732, doi:10.1093/bioinformatics/bth488.

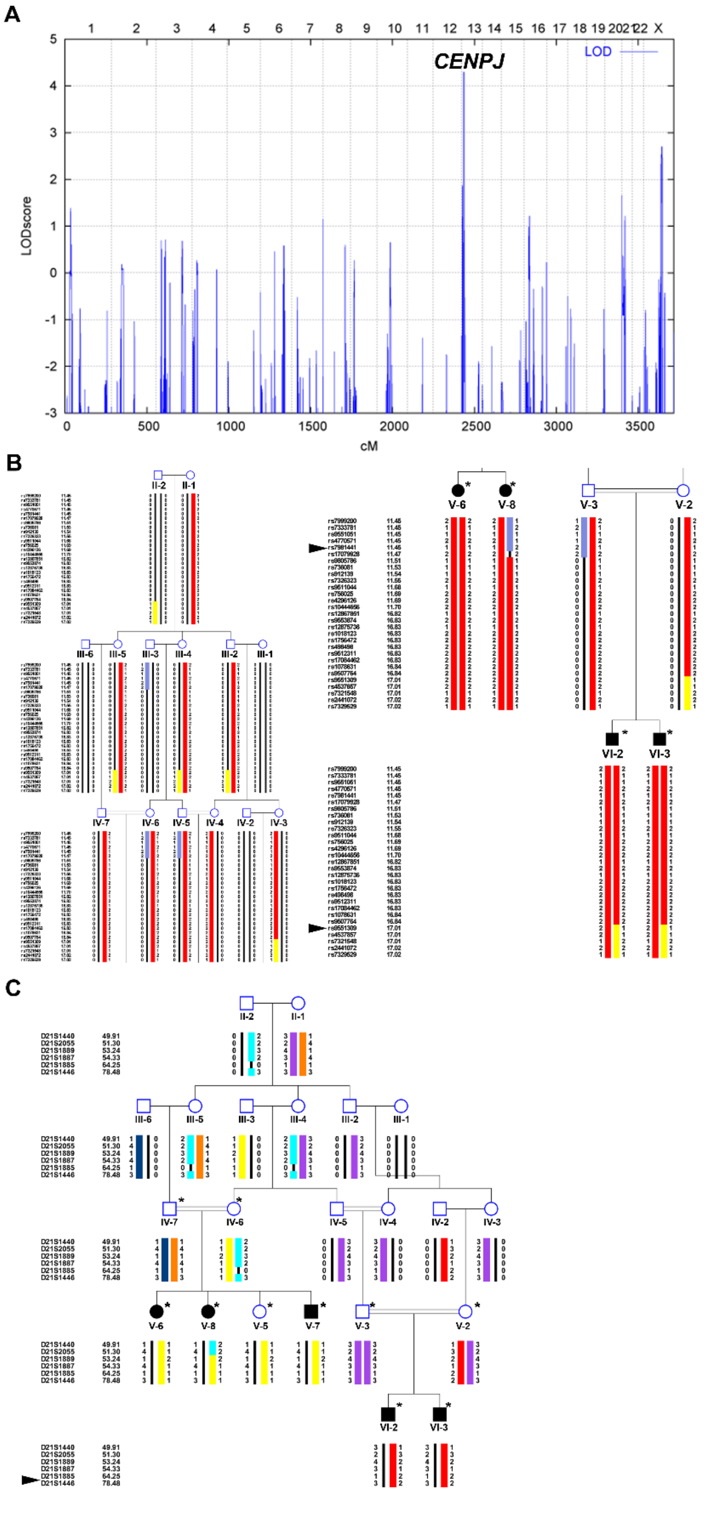

Figure S1. Linkage and haplotype analysis of family 4. **(A)** Genome-wide LOD plot using markers equally spaced by 100 kb. The maximum LOD score of 4.3 was reached on chr 13. The homozygous region covers the *CENPJ* locus. **(B)** Haplotypes generated to show the homozygous region of approximately 2.63 Mb located at chromosome 13 harboring *CENPJ*. The haplotype

drawing is splitted into two panels. Left panel indicates the family members belonging to second, third and fourth generations whereas individuals belonging to fifth and sixth generations are shown in the right panel. Arrowheads show the recombinant markers, rs7981441 (24,075,006 bp) and rs9551309 (26,712,326 bp). Physical positions refer to human genome build GRCh38.p12. **(C)** Haplotypes constructed by genotyping microsatellite markers surrounding *PCNT.* Asterisks denote the affected members used for genotyping. The arrowhead pointing between D21S1885 (chr21:44,759,989-44,960,367 bp) and D21S1446 (chr21:47,937,583-48,129,895 bp) indicates the position of *PCNT* (47,743,976-47,865,682 bp). Physical positions refer to human genome build GRCh37.p13.

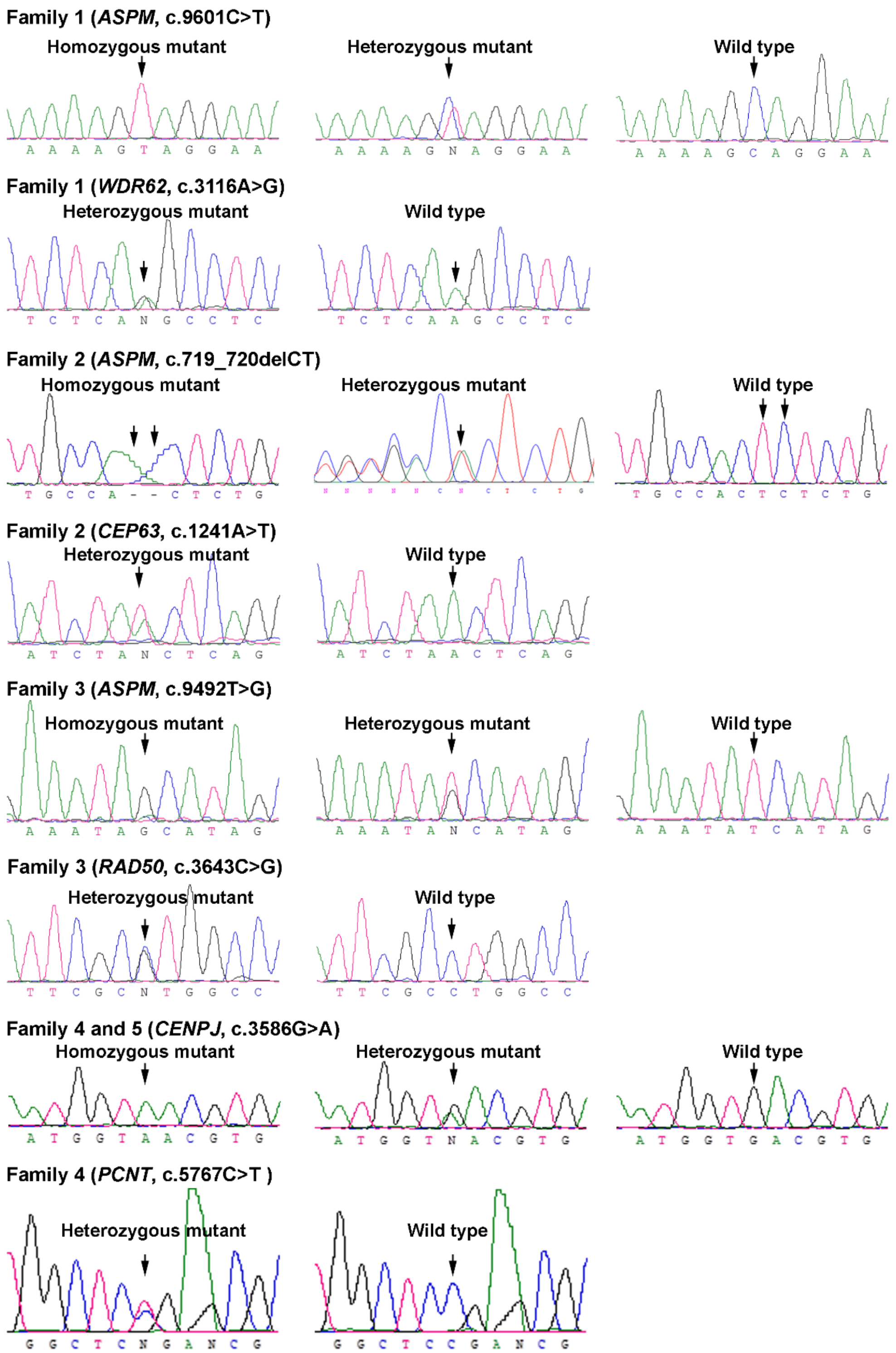

Figure S2. Sanger traces of the identified mutations are shown. For each family, chromatograms of the homozygous disease-causing variations are shown on top, and below are the traces of heterozygous modifying variations. Traces of *ASPM*;c.719_720delCT heterozygous mutant are shown in reverse orientation.

A

WDR62, Ser1106

```
Homo sapiens                 VEASEAEDH-----FFNPRLSISTQFLSSLQKASRFTHTFPPRATQCLVKSPEVKLMDRG
Struthio camelus australis   LKPPEAGEE-EKTLFLNPRLSISARFLSRCQKNSRWVLNAAYPSVRPEKGLPGACWARKP
Bos taurus                   LKAPETEDD-----FFNPRLSISAQFLSRLQKTSRFPHAFPSRLPQHLVKSSEVRLTDLR
Mus musculus                 IKISETEDY-----FFNPRLSISTQFLSRLQKTS----RCPPRLPLHLMKSPEAQPVGQG
Monodelphis domestica        VEPPEMDADSEKSFFLNSRLSMSTQFLSRFQKNSRFAHTFPPTLPLQLVKPLEAVVLRRE
Podarcis muralis             LKAPEEEEE-EAALFQNPRLSISTRFLSSCQKNSRFAAPFLPRLRQHSQAAAADQLAEES
Danio rerio                  LKPTEDHGN---DIFLHPRLSISARFLSRCQKNSSFSRWYPSLFGLTSITLLKYFLSIRK
                             :   :         * :.***:*::* *   * *
```

B

WDR62, Ser1106

```
Macaca mulatta        LGDVEASEAEEYFFNPRLSISTQFLSSLQKASRFTHTLPPRATQHLVKSPEVKPMDQVGS
Cercocebus atys       LGDVEASEAEEYFFNPRLSISTQFLSSLQKASRFTHTLPPRATQHLVKSPEVKPMDQVGS
Homo sapiens          --DVEASEAEDHFFNPRLSISTQFLSSLQKASRFTHTFPPRATQCLVKSPEVKLMDRGGS
Pan troglodytes       LGDVEASEAEDYFFNPRLSISTQFLSSLQKASRFTHTFPPRATQCLVKSPEVKLMDRGGS
Gorilla gorilla       LGDTEASEAEDYFFNPRLSISTQFLSSLQKASRFTHTFPPRATQCLVKSPEVKLMDRGGS
Pongo abelii          --DVEASEAEDYFFNPRLSISTQFLSSLQKASRFTHTFPPRATQRLVKSPEVKLMDRGGS
Callithrix jacchus    LGDTEASEAEDYYFNPRLSISTQFLSSHQKASRFTHTFPSRATQHLVKPPEVKPTDRGRS
Microcebus murinus    LRDMKVSEAEDYYN-PRLSISAQFLSSLRKTSRFPHTFPTRLPQRLVTSPEVKPTARRRS
                        * :.****:::  ******:*****  :*:***.**:*.*  .* **..****    :  *
```

C

Cep63, Thr421

```
Homo sapiens          KMEISHLTQELHQRDITIASTKGSSSDMEKRLRAEMQKAEDKAVEHKEILDQLESLKLEN
Pongo abelii          KMEISHLTQELHQRDITIASTKGSSSDMEKRLRAEMQKAEDKAVEHKEILDQLESLKLEN
Mus musculus          KMEISQLTRELHQRDITIASAKCSSSDMEKQLKAEMQKAEEKAVEHKEILSQLESLKLEN
Rattus norvegicus     KTEISQLTRELHQRDITIASAKCSSSDMERQLKAEMQKAEEKAVEHKEILSQLESLRLEN
Gallus gallus         RKEILRLTQELHQRDITIASAGGSTSDLEQRLRMEIERAERKAVEHRMILVQLETLKLEN
Xenopus laevis        RKEISQLTQELHQRDIRMASSAG--IDWERKIKAERQRAEREAAEHRMSLNALENLRQEN
Danio rerio           RKEVFQLTSELHQRDITIATLTGSTSSIERQLRAEVERAERRASELKVTQVQLETLKLEN
                      : *: :** *******  :*:         . *:::: * ::**  .* * :      **.*: **
```

D

hRAD50, Leu1215

```
Homo sapiens               QKVLASLIIRLALAETFCLNCGIIALDEPTTNLDRENIESLAHALVEIIK
Mus musculus               QKVLASLIIRLALAETFCLNCGILALDEPTTNLDRENIESLAHALVEIIK
Monodelphis domestica      QKVLASLIIRLALAETFCLNCGILALDEPTTNLDRENIESLAHALVEIIK
Gallus gallus              QKVLASLIIRLALAETFCLNCGILALDEPTTNLDRENIESLAHALVEIIK
Xenopus laevis             QKVLASLIIRLALAETFCLNCGILALDEPTTNLDRENIESLAHALVEIIK
Danio rerio                QKVLASLIIRLALAETFCLNCGILALDEPTTNLDRENIESLAHALVEIIK
Caenorhabditis elegans     QKMLASLLIRIALAEVFGGSCSMIALDEPTTNLDESKVEGMAIVLADIIA
Drosophila melanogaster    QRVLASLIIRLALAETFSSNCGVLALDEPTTNLDRANINSLCEALNCIVE
Arabidopsis thaliana       QKVLASLIIRLALAETFCLNCGILALDEPTTNLDGPNSESLAGALLRIME
Saccharomyces cerevisiae   QKVLASIIIRLALSETFGANCGVIALDEPTTNLDEENIESLAKSLHNIIN
                           *::***::**:**:*.*   .*.::**********   :  :.:.   *   *:
```

E

CENP-J, Asp1196

```
Homo sapiens               KVYKNGCRVILFPNGTRKEVSADGKTITVTFFNGDVKQVMP-DQRVIYYYAAAQTTHTTY
Bos taurus                 KVYKNGCHVVLFPNGTRKEVSADGETVTVTFFNGDIKQVMP-DGRVVYYYAATQTAHTTF
Mus musculus               KIYKNGRRVVLFPNGTRKEVSADGKSVTVTFFNGDVKQVMP-DERVVYYYAAAQTTHTTY
Gallus gallus              KVLKNGCHLIFLPNGTWKKVGSDGKTVTITFFNGDVKQIMP-DQTVIYYYADAKTTHTTY
Danio rerio                KMLPDGGRLVVFPNGTRKELSADGQTVKVMFFNGDVKHTMP-DQRVIYYYAEAQTTHITY
Latimeria chalumnae        QVLRSGHRIIVFPNGTRKEVSSDGKTVTVTFFNGDIKQIMP-DQRVIYYYADAKTTHTTY
Xenopus tropicalis         RILRNGYHVIEFPNGTRKEVSADGKSTTVKFFNGDVKQVLG-DQRIIYYYADDQTTHTTY
Monosiga brevicollis       TILPDGTQVIEFPNGTVKKVFADGR-VHVAFFNGDTKETHA-DGRVVYFYQEAGTKHTTL
Octopus bimaculoides       KVYQNGARELIFSNGTRKEMSADGKHIALHFFNGDIKQITA-DQTVIYFYAESETLVTTY
Spizellomyces punctatus    RIYANGIKLVRYSNGTLKEVHPDDELIVIHFTNGDSKTVFA-DGRIVYWYTEQRTLHTTY
Drosophila melanogaster    ITNADGSKDIWYPNGNLKKISADGMNLRMLYFNKDIKETNIREGTVKYYYAETNTWHTSY
                               :*  :  :   .**.  *::  .*.   :  :  :  *  *  *       :    :  *:*     *    :
```

Figure S3. Multiple alignments of short peptide sequences of WDR62, Cep63, hRAD50 and CENP-J from different species. Sites of the mutant residue are colored in red. **(A)** UniProtKB IDs of WDR62 from different organisms are as follows, O43379_HUMAN, XP_009666581 (Ostrich), A0A4W2FHS7_BOBOX, Q3U3T8_MOUSE, F6UN22_MONDO, A0A670J9V7_PODMU and F6NPK9_DANRE. **(B)** UniProtKB and NCBI reference sequences IDs of WDR62 from selected primates have the following IDs, F7CAC3_MACMU, A0A2K5MJV0_CERAT, O43379_HUMAN, H2QG55_PANTR, G3QFQ3_GORGO,

H2NYJ6_PONAB, U3BAJ3_CALJA and XP_012630407. 1_MICMU. **(C)** UniProtKB IDs of Cep63 are Q96MT8_HUMAN, Q5NVN6_PONAB, Q3UPP8_MOUSE, Q4KLY0_RAT, P0CB05_CHICK, B9V5F5_XENLA and Q6PGZ0_DANRE. **(D)** UniProtKB and NCBI reference sequence IDs of hRAD50 are Q92878_HUMAN, P70388_MOUSE, XP_007473506. 1_MONDO, Q5W4T6_CHICK, A0A1L8GXM0_XENLA, F8W411_DANRE, O44199_CAEEL, Q9W252_DROME, Q9SL02_ARATH and P12753_YEAST. **(E)** UniProtKB IDs of CENP-J are as follows, Q9HC77_HUMAN, E1BL95_BOVIN, Q569L8_MOUSE, E1C0R8_CHICK, E7FCY1_DANRE, H2ZX87_LATCH, F7C9Z3_XENTR, A9V5C9_MONBE, A0A0L8G7N5_OCTBM, A0A0L0HNY0_SPIPN and Q9VI72_DROME.

Figure S4. Immunoblot showing the reduced amount of CENP-J in both patients (VI-2 and V-8) compared to wild type.

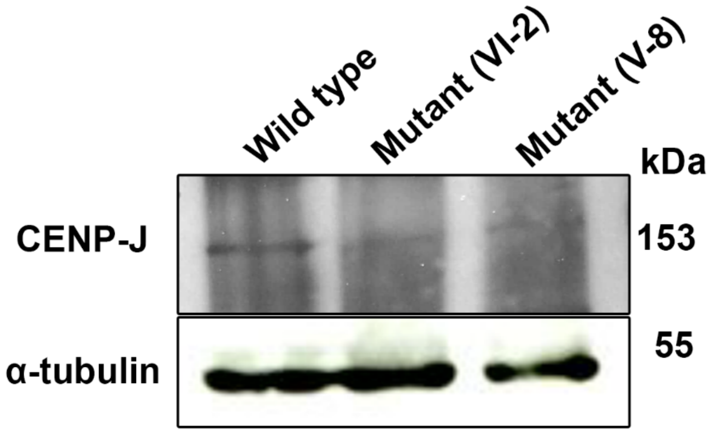

Supplement: Supplementary file 1 [file genes-12-00731-s001.zip › genes-1206322-supplementary.pdf]
